# Neuropsychiatric symptoms in genetic frontotemporal dementia: developing a new module for Clinical Rating Scales

**DOI:** 10.1136/jnnp-2022-330152

**Published:** 2023-01-10

**Authors:** Kiran Samra, Amy Macdougall, Georgia Peakman, Arabella Bouzigues, Martina Bocchetta, David M Cash, Caroline V Greaves, Rhian S Convery, John C van Swieten, Lize C Jiskoot, Harro Seelaar, Fermin Moreno, Raquel Sánchez-Valle, Robert Laforce, Caroline Graff, Mario Masellis, Maria Carmela Tartaglia, James B Rowe, Barbara Borroni, Elizabeth Finger, Matthis Synofzik, Daniela Galimberti, Rik Vandenberghe, Alexandre de Mendonca, Christopher R Butler, Alexander Gerhard, Simon Ducharme, Isabelle Le Ber, Pietro Tiraboschi, Isabel Santana, Florence Pasquier, Johannes Levin, Markus Otto, Sandro Sorbi, Jonathan D Rohrer, Lucy L Russell, the Genetic FTDInitiative (GENFI), Annabel Nelson

**Affiliations:** 1 Dementia Reseach Centre, Department of Neurodegenerative Disease, UCL Queen Square Institute of Neurology, London, UK; 2 London School of Hygiene & Tropical Medicine, London, UK; 3 Centre for Medical Image Computing, University College London, London, UK; 4 Neurology, Erasmus MC, Rotterdam, The Netherlands; 5 Neurology, Erasmus MC Alzheimer Centre, Rotterdam, The Netherlands; 6 Cognitive Disorders Unit, Department of Neurology, Donostia University Hospital Gipuzkoa Building, San Sebastian, Spain; 7 Alzheimer's Disease and Other Cognitive Disorders Unit, Neurology Service, Hospital Clinic de Barcelona, Barcelona, Spain; 8 Interdisciplinary Memory Clinic, Department of Neurological Sciences, Laval University, Quebec, Quebec, Canada; 9 Center for Alzheimer Research, Division of Neurogeriatrics, Department of Neurobiology, Care Sciences and Society, Karolinska Institutet, Stockholm, Sweden; 10 Unit for Hereditary Dementias, Theme Aging, Karolinska University Hospital, Solna, Sweden; 11 Neurology, Sunnybrook Health Sciences Centre, Toronto, Ontario, Canada; 12 Tanz Centre for Research in Neurodegenerative Disease, University of Toronto, Toronto, Ontario, Canada; 13 Department of Clinical Neurosciences, University of Cambridge, Cambridge, UK; 14 Centre for Ageing Brain and Neurodegenerative Disorders, Neurology Unit, Department of Clinical and Experimental Sciences, University of Brescia, Brescia, Italy; 15 Clinical Neurological Sciences, University of Western Ontario, London, Ontario, Canada; 16 Dept. of Neurodegenerative Diseases, Eberhard Karls University Tubingen Hertie Institute for Clinical Brain Research, Tubingen, Germany; 17 Department of Neurological Sciences, Fondazione IRCCS Ca' Granda Ospedale Maggiore Policlinico, Milan, Italy; 18 Laboratory for Cognitive Neurology, Department of Neurosciences, KU Leuven, Leuven, Belgium; 19 Neurology Service, KU Leuven University Hospitals Leuven, Leuven, Belgium; 20 Faculty of Medicine, University of Lisbon, Lisboa, Portugal; 21 Nuffield Department of Clinical Neurosciences, University of Oxford, Oxford, UK; 22 Department of Brain Sciences, Imperial College London, London, UK; 23 Division of Neuroscience and Experimental Psychology, The University of Manchester, Manchester, UK; 24 Departments of Geriatric Medicine and Nuclear Medicine, University of Duisburg-Essen, Duisburg, Germany; 25 McConnell Brain Imaging Centre, Department of Neurology & Neurosurgery, Montreal Neurological Institute and Hospital, Montreal, Québec, Canada; 26 Douglas Mental Health University Institute, Department of Psychiatry, McGill University, Montreal, Québec, Canada; 27 Inserm U1127, CNRS UMR 7225, FrontLab - Reference Centre for Rare or Early Dementias, IM2A, Département de Neurologie, Hôpital Universitaire Pitié Salpêtrière, Sorbonne Université, Paris Brain Institute – Institut du Cerveau – ICM, Paris, France; 28 National Reference Center On Rare Dementias, Groupe Hospitalier La Pitié Salpêtrière-Charles Foix, Paris, France; 29 Division of Neurology V and Neuropathology, Foundation IRCCS Carlo Besta Neurological Institute, Milano, Italy; 30 Neurology, Hospital and University Centre of Coimbra, Coimbra, Portugal; 31 Centre for Neuroscience and Cell Biology (CNC).IBILI, University of Coimbra, Coimbra, Portugal; 32 Inserm U1171, University of Lille, Lille, France; 33 Memory Clinic, Neurology, CHU Lille, Lille, France; 34 German Center for Neurodegenerative Diseases (DZNE), DZNE, Bonn, Germany; 35 Department of Neurology, Ludwig Maximilians University Munich, Munchen, Germany; 36 Department of Neurology, University of Ulm, Ulm, Germany; 37 Neurosciences Drugs and Child Health, University of Florence, Firenze, Italy; 38 IRCCS Firenze, Fondazione Don Carlo Gnocchi Onlus, Firenze, Italy

**Keywords:** FRONTOTEMPORAL DEMENTIA, GENETICS, NEUROPSYCHIATRY

## Abstract

**Background:**

Current clinical rating scales in frontotemporal dementia (FTD) often do not incorporate neuropsychiatric features and may therefore inadequately measure disease stage.

**Methods:**

832 participants from the Genetic FTD Initiative (GENFI) were recruited: 522 mutation carriers and 310 mutation-negative controls. The standardised GENFI clinical questionnaire assessed the frequency and severity of 14 neuropsychiatric symptoms: visual, auditory, and tactile hallucinations, delusions, depression, anxiety, irritability/lability, agitation/aggression, euphoria/elation, aberrant motor behaviour, hypersexuality, hyperreligiosity, impaired sleep, and altered sense of humour. A principal component analysis (PCA) was performed to identify key groupings of neuropsychiatric and behavioural items in order to create a new neuropsychiatric module that could be used as an addition to the Clinical Dementia Rating (CDR) plus National Alzheimer’s Coordinating Center Behaviour and Language Domains (NACC FTLD) rating scale.

**Results:**

Overall, 46.4% of mutation carriers had neuropsychiatric symptoms (51.6% *C9orf72*, 40.8% *GRN*, 46.6% *MAPT*) compared with 24.5% of controls. Anxiety and depression were the most common in all genetic groups but fluctuated longitudinally and loaded separately in the PCA. Hallucinations and delusions loaded together, with the remaining neuropsychiatric symptoms loading with the core behavioural features of FTD. These results suggest using a single ‘psychosis’ neuropsychiatric module consisting of hallucinations and delusions. Adding this to the CDR plus NACC FTLD, called the CDR plus NACC FTLD-N, leads to a number of participants being scored more severely, including those who were previously considered asymptomatic now being scored as prodromal.

**Conclusions:**

Neuropsychiatric symptoms occur in mutation carriers at all disease stages across all three genetic groups. However, only psychosis features provided additional staging benefit to the CDR plus NACC FTLD. Inclusion of these features brings us closer to optimising the rating scale for use in trials.

**WHAT IS ALREADY KNOWN ON THIS TOPIC**

- Neuropsychiatric features are common in sporadic and genetic forms of frontotemporal dementia (FTD), particularly the behavioural variant.

**WHAT THIS STUDY ADDS**

- Neuropsychiatric symptoms occur early on in FTD, before individuals become symptomatic. There are three main groups: affective symptoms (anxiety and depression), ‘psychosis’ symptoms (hallucinations and delusions) and other neuropsychiatric symptoms that formed a group with the core behavioural criteria of FTD. The affective symptoms were common in controls as well as mutation carriers and fluctuated longitudinally, so were excluded from the scale. The psychosis symptoms therefore formed the neuropsychiatric component that was added to the Clinical Dementia Rating plus National Alzheimer’s Coordinating Center Behaviour and Language Domains, resulting in individuals being scored more severely.

**HOW THIS STUDY MIGHT AFFECT RESEARCH, PRACTICE OR POLICY**

- Future revisions of FTD clinical rating scales should include neuropsychiatric symptoms to capture the entire spectrum of disease. This will in turn optimise selection of individuals into therapeutic trials.

## Introduction

Frontotemporal dementia (FTD) is a heterogeneous neurodegenerative disorder that commonly presents with either personality change (behavioural variant FTD, bvFTD) or speech and language difficulties (primary progressive aphasia, PPA). The core behavioural symptoms recognised in the diagnostic criteria of bvFTD are disinhibition, apathy, loss of sympathy or empathy, ritualistic-compulsive behaviour and appetite change.^1^ However, a number of changes in personality and behaviour that are seen in people with FTD do not fit into these core criteria, and it has become increasingly recognised that there is a wider set of neuropsychiatric symptoms that occur in bvFTD,^2^ and to a lesser extent in PPA,^3^ many of which overlap with those seen in primary psychiatric disorders like major depressive disorder, bipolar disorder and schizophrenia.

While studies have highlighted the presence of neuropsychiatric symptoms in sporadic forms of FTD,^2 3^ they seem to be particularly prevalent in genetic FTD^4–8^ caused by mutations in progranulin (*GRN*), chromosome 9 open reading frame 72 (*C9orf72*) and microtubule-associated protein tau (*MAPT*).^9^ However, such symptoms are not included in either of the main clinical rating scales for FTD, the Clinical Dementia Rating Dementia Staging Instrument plus National Alzheimer’s Coordinating Center Behaviour and Language Domains (CDR plus NACC FTLD),^10–12^ or the FTD Rating Scale.^13 14^ Inclusion of a neuropsychiatric domain in rating scales is particularly relevant to people with FTD and their caregivers, as such symptoms are highly associated with morbidity and caregiver burden.^15 16^ Furthermore, exclusion of a neuropsychiatric domain risks inappropriately rating people at an earlier, less severe stage of the disease than they actually are.

This study, therefore, aims to understand how best to include neuropsychiatric symptoms as an additional module to the CDR plus NACC FTLD, a scale commonly used in current clinical trials of FTD. In particular, the study aims to investigate whether neuropsychiatric symptoms are best assessed as part of, or separately to, the core behavioural symptoms that are currently included in the behaviour module of the CDR plus NACC FTLD. Better understanding of an individual’s disease stage and their progression by incorporating neuropsychiatric symptoms into the scale will enhance therapeutic trial design and improve identification of treatment response.

## Methods

### Participants

Participants were recruited from the fifth data freeze of the Genetic FTD Initiative (GENFI) study between 20 January 2012 and 30 May 2019, including sites in the UK, Canada, Belgium, France, Germany, Italy, the Netherlands, Portugal, Spain and Sweden.

The standardised GENFI clinical assessment included a clinical history and neurological examination, neuropsychometric assessment and the CDR plus NACC FTLD.^10^ Mutation carriers were classified into asymptomatic, prodromal or symptomatic if they scored 0, 0.5 or ≥1, respectively, using the CDR plus NACC FTLD global score. To investigate neuropsychiatric features, we reviewed all mutation carriers recruited in the study at baseline visit, including 221 *C9orf72*, 213 *GRN* and 88 *MAPT* mutation carriers. Of these 522 mutation carriers, the CDR plus NACC FTLD global score was 0 in 55.7%, 0.5 in 15.7% and ≥1 in 28.5%. Participants were also separately judged by a clinician whether they were felt to be symptomatic. In this group, 109 had bvFTD (20.9% of the mutation carriers studied),^1^ and 26 had PPA (5.0%).^17^ Additionally, 17 had amyotrophic lateral sclerosis (ALS) or FTD-ALS (3.3%),^18 19^ and 5 had a parkinsonian disorder (1.0%).^20 21^ The control group consisted of healthy non-mutation carriers from the GENFI cohort with a CDR plus NACC FTLD global score of 0 or 0.5 (310 in total). Demographics are shown in table 1.

**Table 1:** Demographics, clinical scores and frequency of neuropsychiatric symptoms for all mutation carriers (*C9orf72, GRN, MAPT*) and healthy controls

CDR plus NACC FTLD global score	Controls	All mutation carriers				*C9orf72*				*GRN*				*MAPT*			
		All	0	0.5	≥1	All	0	0.5	≥1	All	0	0.5	≥1	All	0	0.5	≥1
No of participants	310	522	291	82	149	221	112	37	72	213	130	31	52	88	49	14	25
% male	44	44	38	41	58	49	41	41	65	39	35	48	46	45	41	29	64
Age (years)	46.0 (12.7)	50.1 (13.7)	44.2 (11.9)	49.7 (12.3)	62.0 (9.2)	51.2 (13.6)	44.5 (11.7)	49.4 (11.2)	62.7 (9.3)	51.0 (13.6)	45.8 (12.2)	51.8 (13.2)	63.5 (7.7)	45.3 (13.1)	39.2 (10.4)	45.7 (12.6)	57.0 (10.1)
Education (years)	14.5 (3.3)	13.9 (3.4)	14.5 (3.2)	13.9 (3.1)	12.8 (3.7)	13.9 (3.2)	14.4 (3.0)	14.1 (2.6)	13.1 (3.7)	13.9 (3.7)	14.7 (3.4)	14.0 (4.0)	11.9 (3.5)	14.1 (3.3)	14.4 (3.3)	13.5 (2.4)	13.6 (3.8)
MMSE	29.3 (1.0)	27.1 (5.3)	29.3 (1.0)	28.5 (2.2)	21.9 (7.6)	27.2 (4.7)	29.1 (1.2)	28.5 (2.1)	23.4 (6.6)	26.9 (6.0)	29.4 (0.9)	28.5 (2.4)	19.3 (8.4)	27.4 (5.1)	29.5 (0.8)	28.2 (2.3)	22.8 (7.7)
CDR plus NACC FTLD Global score	0.1 (0.2)	0.6 (1.0)	0.0 (0.0)	0.5 (0.0)	2.0 (0.8)	0.8 (1.0)	0.0 (0.0)	0.5 (0.0)	2.1 (0.8)	0.5 (0.9)	0.0 (0.0)	0.5 (0.0)	1.9 (0.8)	0.6 (0.9)	0.0 (0.0)	0.5 (0.0)	1.9 (0.8)
CDR plus NACC FTLD Sum of Boxes	0.2 (0.4)	3.1 (5.5)	0.0 (0.0)	1.1 (0.8)	10.3 (5.8)	3.8 (6.0)	0.0 (0.0)	1.1 (0.8)	10.9 (5.7)	2.5 (5.1)	0.0 (0.0)	1.0 (0.8)	9.8 (6.0)	2.9 (5.3)	0.0 (0.0)	1.1 (0.8)	9.7 (5.8)
Neuropsychiatric symptoms (%)	24.5	46.4	18.0	70.7	89.3	51.6	19.6	70.3	91.7	40.8	16.9	71.0	82.7	46.6	14.3	71.4	96.0

Age, education, MMSE and Clinical Rating Scale scores are shown as mean (SD).

CDR, Clinical Dementia Rating; MMSE, Mini-Mental State Examination; NACC FTLD, National Alzheimer’s Coordinating Center Behaviour and Language Domains.

### Neuropsychiatric symptoms

Neuropsychiatric symptoms were assessed using the GENFI neuropsychiatric symptom scale (online supplemental table 1).^4^ This contains 14 symptoms scored as per the CDR scale, that is, 0=asymptomatic, 0.5=questionable/very mild, 1=mild, 2=moderate and 3=severe: visual, auditory and tactile hallucinations, delusions, depression, anxiety, irritability/lability, agitation/aggression, euphoria/elation, aberrant motor behaviour, hypersexuality, hyperreligiosity, impaired sleep and altered sense of humour.

10.1136/jnnp-2022-330152.supp1 Supplementary data

We also wanted to explore the overlap of these neuropsychiatric symptoms with the core behavioural symptoms described in the international consensus criteria for bvFTD.^1^ In the GENFI behavioural symptom scale, seven symptoms are measured: disinhibition, apathy, loss of sympathy/empathy, ritualistic/compulsive behaviour and hyperorality and appetite changes, as well as poor response to social/emotional cues and inappropriate trusting behaviour.

### Statistical analysis

All statistical analyses were performed using Stata/MP V.16.1 unless otherwise specified. All graphs were produced using GraphPad Prism V.9 except for the Sankey diagrams which were made using SankeyMATIC. Demographics were compared between groups using either linear regression (age and education) or a χ^2^ test (sex), and age- and sex-adjusted linear regressions to compare the Mini-Mental State Examination (MMSE) and CDR plus NACC FTLD. Frequency and severity of individual neuropsychiatric symptoms were compared between groups using ordinal logistic regressions adjusting for age and sex, except for the comparisons of disease group versus controls for severity (as there was minimal variation from zero for the control group) and frequency (when the controls scored zero) where linear regressions adjusting for age and sex were used, with 95% bias-corrected bootstrapped confidence intervals with 2000 repetitions.

Principal component analysis (PCA) was performed in R V.4.1.2^22^ in order to see whether it was possible to combine the multiple items in the neuropsychiatric symptom list into a smaller number of variables with minimal loss of information that could then form one or more modules to incorporate into a rating scale. Components with an eigenvalue greater than one were selected and the varimax rotation was used. An initial PCA was performed looking only at neuropsychiatric symptoms in all mutation carriers and in each of the genetic mutation subgroups (*C9orf72*, *GRN*, *MAPT*). A further PCA included behavioural as well as neuropsychiatric symptoms in the analyses was also performed. Items within a component with loadings closest to +1.00 or −1.00 were interpreted as loading strongly onto that factor, while those nearest to zero were considered as loading weakly.

As mood symptoms are common in the general population and can fluctuate with time, both with and without treatment (whether pharmacological or talking therapies), we decided to additionally perform a subanalysis of depression and anxiety and how these features change in frequency and severity over time. Descriptive differences were reported for each time point over three visits.

### Rating scale analysis

Finally, we investigated the addition of a neuropsychiatric module to the CDR plus NACC FTLD rating scale. We compared this new scale (termed CDR plus NACC FTLD-N) with the original CDR plus NACC FTLD. We also developed a new version of the behavioural module based on the PCA findings and compared this (termed CDR plus NACC FTLD-N-B+) with the original CDR plus NACC FTLD.

## Results

### Demographics

No significant differences were seen between the mutation groups in years of education, but the overall mutation carrier group and *C9orf72* mutation carriers had, on average, significantly fewer years of education than controls (p=0.024 and p=0.048, respectively). *C9orf72* and *GRN* mutation carriers were significantly older than controls (both p<0.001) and *MAPT* mutation carriers (p<0.001 and p=0.001 respectively), while the *C9orf72* group contained more males than the *GRN* group (χ^2^=3.91, p=0.048) (table 1).

### Disease severity

The MMSE and CDR plus NACC FTLD Sum of Boxes scores were significantly different to controls in each genetic group overall (table 1). There were no significant differences between the genetic groups (all mutation carriers combined) apart from for the CDR plus NACC FTLD which was higher in *C9orf72* compared with the *GRN* group (both global and sum of boxes scores, p<0.05).

### Frequency and severity of neuropsychiatric symptoms in the GENFI cohort

Neuropsychiatric symptoms were reported in 46.4% of all mutation carriers: 51.6% of the *C9orf72* group, 40.8% of the *GRN* group and 46.6% of the *MAPT* group. In comparison, 24.5% of controls were reported as showing symptoms. Stratifying by CDR plus NACC FTLD, neuropsychiatric symptoms occurred in 18.0% of mutation carriers with a global score of 0 (19.6% *C9orf72*, 16.9% *GRN*, 14.3% *MAPT*), 70.7% of mutation carriers with a global score of 0.5 (70.3% *C9orf72*, 71.0% *GRN*, 71.4% *MAPT*) and 89.3% of mutation carriers with a global score≥1 (91.7% *C9orf72*, 82.7% *GRN*, 96.0% *MAPT*) (table 1).

**Figure 1:**
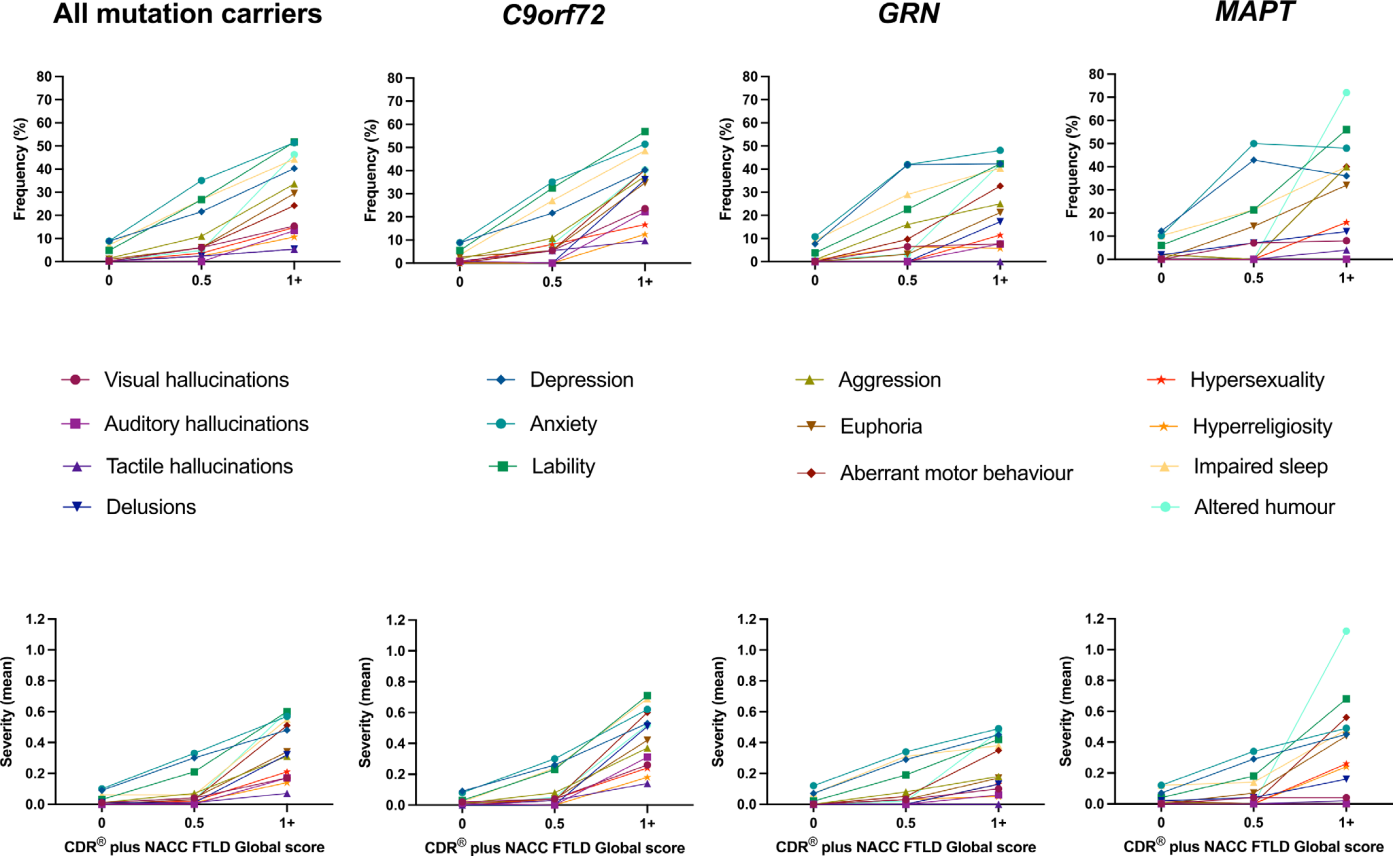
Frequency (top panel) and severity (bottom panel) of symptoms in the GENFI neuropsychiatric symptom scale in all mutation carriers when asymptomatic (CDR plus NACC FTLD global score of 0), prodromal (score of 0.5) and symptomatic (score of 1+). Frequency and severity of symptoms in the individual genetic groups (*C9orf72, GRN* and *MAPT*) are shown similarly to the right of the combined mutation carrier group. CDR, Clinical Dementia Rating; GENFI, Genetic FTD Initiative; NACC FTLD, National Alzheimer’s Coordinating Center Behaviour and Language Domains.

When looking at the individual symptoms in the combined mutation carrier group, anxiety was the most frequent and severe neuropsychiatric symptom, followed by depression, impaired sleep and irritability/lability. Stratifying this group by CDR plus NACC FTLD, all of the neuropsychiatric symptoms were significantly more impaired (ie, more frequent and more severe) than controls when the global score was ≥1 (figure 1, table 2, online supplemental table 2): lability/irritability (frequency 51.7%, mean (SD) severity 0.60 (0.79)), anxiety (49.7%, 0.57 (0.73)), altered sense of humour (46.3%, 0.60 (0.84)), impaired sleep (44.3%, 0.55 (0.76)) and depression (40.3%, 0.48 (0.73)), were the most frequent and severe. At the prodromal stage (CDR plus NACC FTLD global score of 0.5), anxiety (40.2%, 0.33 (0.47)), depression (32.9%, 0.30 (0.52)), irritability/lability (26.8%, 0.21 (0.42)) and impaired sleep (26.8%, 0.25 (0.49)) remained the most frequent and severe symptoms, but visual hallucinations, agitation/aggression, euphoria/elation, aberrant motor behaviour, hypersexuality and altered sense of humour all occurred significantly more frequently than controls (table 2). At a CDR plus NACC FTLD global score of 0 none of the symptoms were significantly more frequent or severe than controls, but anxiety, irritability/lability and impaired sleep occurred less frequently.

**Table 2:** Percentage frequency of individual neuropsychiatric symptoms in controls and mutation carriers

	Controls	All mutation carriers			*C9orf72*			*GRN*			*MAPT*		
		CDR 0	CDR 0.5	CDR ≥1	CDR 0	CDR 0.5	CDR ≥1	CDR 0	CDR 0.5	CDR ≥1	CDR 0	CDR 0.5	CDR ≥1
Visual hallucinations	0.3	0.3	**6.1**	**15.4**	0.9	**5.4**	**23.6**	0.0	**6.5**	**7.7**	0.0	**7.1**	**8.0**
Auditory hallucinations	1.3	0.3	0.0	**13.4**	0.9	0.0	**22.2**	0.0	0.0	**7.7**	0.0	0.0	0.0
Tactile hallucinations	0.0	0.3	2.4	**5.4**	0.9	5.4	**9.7**	0.0	0.0	0.0	0.0	0.0	4.0
Delusions	1.0	0.7	1.2	**25.5**	0.9	0.0	**36.1**	0.0	0.0	**17.3**	2.0	7.1	**12.0**
Depression	12.9	8.9	**32.9**	**40.3**	8.9	21.6	**40.3**	7.7	**41.9**	**42.3**	12.2	**42.9**	**36.0**
Anxiety	15.5	*10.0*	**40.2**	**49.7**	8.9	**35.1**	**51.4**	10.8	**41.9**	**48.1**	10.2	**50.0**	**48.0**
Irritability/lability	11.9	*4.8*	**26.8**	**51.7**	5.4	**32.4**	**56.9**	*3.8*	22.6	**42.3**	6.1	21.4	**56.0**
Agitation/aggression	2.6	1.4	**11.0**	**33.6**	1.8	**10.8**	**37.5**	0.8	**16.1**	**25.0**	2.0	*0.0*	**40.0**
Euphoria/elation	0.3	1.0	**6.1**	**29.5**	**2.7**	**5.4**	**34.7**	0.0	3.2	**21.2**	0.0	**14.3**	**32.0**
Aberrant motor behaviour	1.3	0.3	**6.1**	**24.2**	*0.0*	5.4	**40.3**	*0.0*	**9.7**	**32.7**	2.0	0.0	**40.0**
Hypersexuality	0.3	0.0	**3.7**	**14.8**	0.0	**8.1**	**16.7**	0.0	0.0	**11.5**	0.0	0.0	**16.0**
Hyperreligiosity	0.0	0.3	2.4	**10.7**	0.0	0.0	**12.5**	0.8	6.5	5.8	0.0	0.0	**16.0**
Impaired sleep	13.2	*7.2*	**26.8**	**44.3**	*3.6*	**27.0**	**48.6**	9.2	**29.0**	**40.4**	10.2	21.4	**40.0**
Altered sense of humour	0.6	0.3	**4.9**	**46.3**	0.0	**8.1**	**40.3**	0.8	3.2	**42.3**	0.0	0.0	**72.0**

Number of cases is as per table 1. Bold items are significantly more frequent than controls and italicised items are significantly less frequent than controls (p<0.05). Other differences are shown as *significantly more frequent compared with *GRN*, †significantly more frequent compared with *MAPT* and ‡significantly more frequent compared with *C9orf72* (p<0.05). See online supplemental file 1 for similar analysis of severity.

Stratifying by genetic group, all symptoms were significantly more frequent and severe in the symptomatic (CDR plus NACC FTLD≥1) *C9orf72* mutation carriers with the majority of symptoms also significantly more frequent (and in most cases more severe) in the symptomatic *GRN* and *MAPT* groups (table 2, online supplemental table 2). Comparing groups, hallucinations and delusions were more frequent and severe in the *C9orf72* group: visual hallucinations frequency 23.6%, severity 0.26 (0.58) vs 7.7%, 0.10 (0.36) in *GRN* mutation carriers and 8.0%, 0.06 (0.22) in *MAPT* mutation carriers; auditory hallucinations 22.2%, 0.31 (0.71) vs 7.7%, 0.06 (0.21) and 0.0%, 0.00 (0.00); tactile hallucinations 9.7%, 0.14 (0.53) vs 0.0%, 0.00 (0.00) and 4.0%, 0.02 (0.10); delusions 36.1%, 0.51 (0.81) vs 17.3%, 0.13 (0.36) and 12.0%, 0.16 (0.47). In contrast, altered sense of humour was more frequent and severe in symptomatic *MAPT* mutation carriers compared with the other two groups (72.0%, 1.12 (1.07) vs 40.3%, 0.52 (0.79) in *C9orf72* and 42.3%, 0.47 (0.70) in *GRN*). When the CDR plus NACC FTLD was 0.5 anxiety (*C9orf72* 35.1%, *GRN* 41.9%, *MAPT* 50.0%), depression (*C9orf72* 21.6%, *GRN* 41.9%, *MAPT* 42.9%), impaired sleep (*C9orf72* 27.0%, *GRN* 29.0%, *MAPT* 21.4%) and irritability/lability (*C9orf72* 32.4%, *GRN* 22.6%, *MAPT* 21.4%) were the most frequent symptoms. However, visual hallucinations were more common in all three groups (*C9orf72* 5.4%, *GRN* 6.5%, *MAPT* 7.1%) and agitation/aggression (in *C9orf72* and *GRN*), euphoria/elation (in *C9orf72* and *MAPT*), aberrant motor behaviour (in *GRN*), hypersexuality (in *C9orf72*) and altered sense of humour (in *C9orf72*) all occurred more frequently than in controls. Only euphoria/elation was more frequent (in the *C9orf72* group) at a CDR plus NACC FTLD of 0 with no other symptoms more frequent or severe than controls (figure 1, table 2, online supplemental table 2).

### Principal component analysis

**Figure 2:**
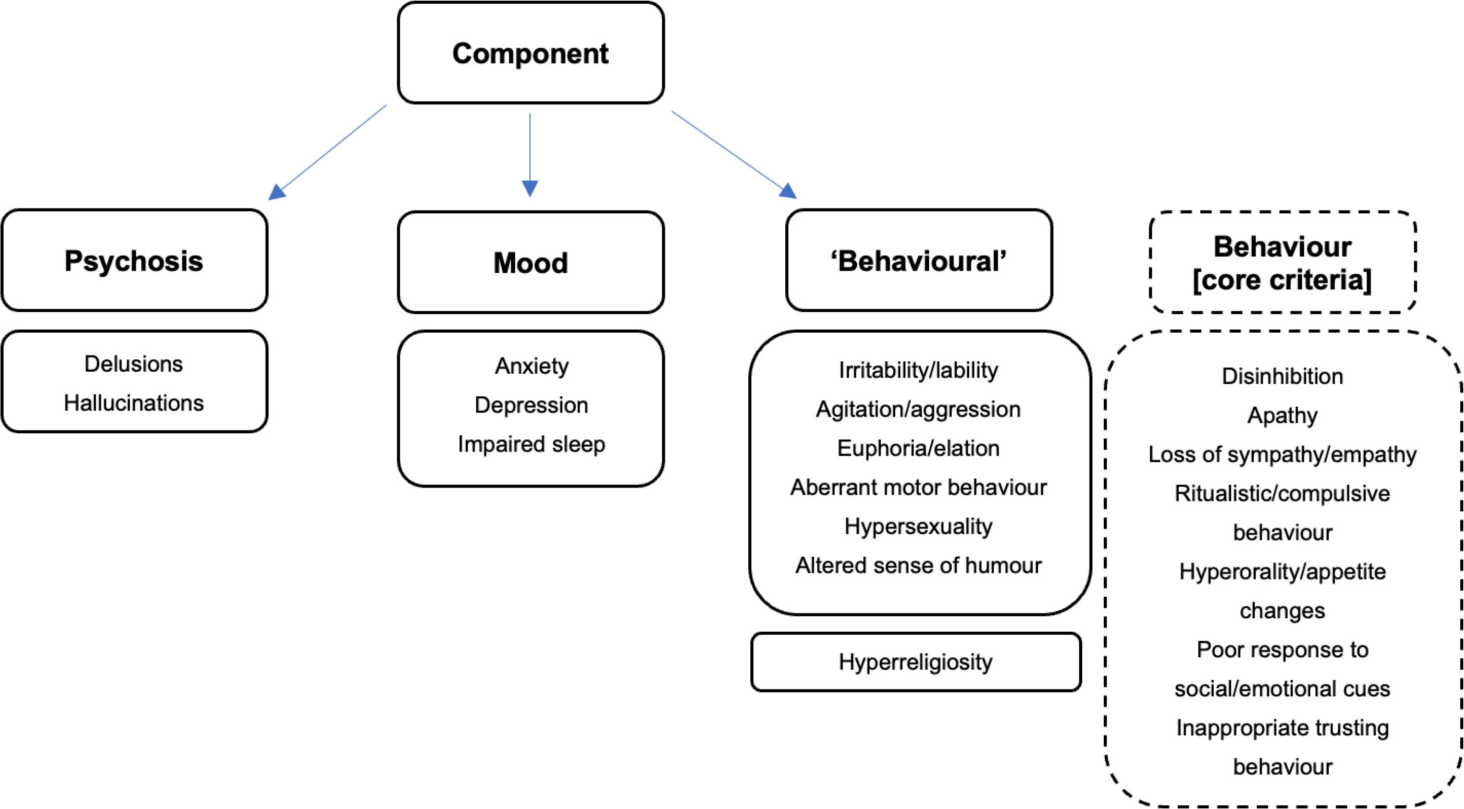
Visual representation of the principal component analysis (PCA) results. The PCA of the neuropsychiatric symptom scale revealed four components, named for the items which loaded most strongly: ‘psychosis’, ‘mood’ and ‘behavioural’, with a fourth component of hyperreligiosity. The dotted boxes represent the finding that in a further PCA which included the core behavioural symptoms of FTD these items loaded alongside the ‘behavioural’ features of the neuropsychiatric symptom scale. FTD, frontotemporal dementia.

PCA of the neuropsychiatric symptoms loaded on to four main components, which cumulatively explained 28%, 49%, 69% and 81% of the variation in the data respectively. Component one showed loading mainly of the non-psychosis and non-affective (‘behavioural’) symptoms, component two strongly loaded visual and auditory hallucinations and delusions (ie, features of ‘psychosis’), component three loaded depression, anxiety and impaired sleep (ie, ‘mood’ symptoms), and component four loaded hyperreligiosity (table 3, figure 2). PCA of the neuropsychiatric symptoms in the individual genetic groups produced similar results (online supplemental table 3a).

**Table 3:** Principal component analysis of neuropsychiatric symptoms in all mutation carriers

Component	1	2	3	4
Visual hallucinations	0.19	0.88	0.22	0.00
Auditory hallucinations	0.25	0.88	0.27	0.00
Tactile hallucinations	0.38	0.24	0.32	−0.85
Delusions	0.41	0.73	0.28	0.00
Depression	0.00	0.19	0.85	0.00
Anxiety	0.25	0.22	0.80	0.00
Irritability/lability	0.68	0.11	0.58	0.00
Agitation/aggression	0.75	0.10	0.40	0.00
Euphoria/elation	0.84	0.26	0.13	0.00
Aberrant motor behaviour	0.66	0.49	0.16	0.11
Hypersexuality	0.77	0.22	0.20	−0.14
Hyperreligiosity	0.32	0.36	0.22	0.83
Impaired sleep	0.28	0.31	0.72	0.00
Altered sense of humour	0.74	0.39	0.00	0.34
Cumulative variance	0.28	0.49	0.69	0.81

When adding the core behavioural symptoms to the PCA, a similar result was found with four components, but in this analysis all the core behavioural symptoms loaded with the other ‘behavioural’ (ie, non-psychosis, non-affective) symptoms from the neuropsychiatric symptom scale (apart from hyperreligiosity, which as previously, loaded separately) (table 4, online supplemental table 3b, figure 2).

**Table 4:** Principal component analysis of combined neuropsychiatric and behavioural symptoms in all mutation carriers

Component	1	2	3	4
Visual hallucinations	0.13	0.89	0.21	−0.21
Auditory hallucinations	0.32	0.90	0.11	0.00
Tactile hallucinations	0.00	0.00	−0.89	0.26
Delusions	0.43	0.74	−0.15	0.20
Depression	−0.76	−0.11	0.00	0.35
Anxiety	−0.68	−0.20	0.00	0.47
Irritability/lability	0.31	−0.61	−0.19	0.58
Agitation/aggression	0.59	−0.56	−0.22	0.26
Euphoria/elation	0.78	−0.33	−0.14	−0.39
Aberrant motor behaviour	0.85	0.14	0.18	0.00
Hypersexuality	0.60	−0.35	−0.43	−0.25
Hyperreligiosity	0.38	0.19	0.83	0.00
Impaired sleep	−0.37	0.00	−0.18	0.78
Altered sense of humour	0.85	0.00	0.40	−0.16
Disinhibition	0.94	0.00	−0.21	0.00
Apathy	0.88	0.23	0.16	0.00
Loss of sympathy/empathy	0.93	0.20	0.17	0.00
Ritualistic/compulsive behaviour	0.93	0.00	0.00	0.00
Hyperorality and appetite changes	0.92	0.19	0.27	0.00
Poor response to social/emotional cues	0.96	0.12	0.00	0.00
Inappropriate trusting behaviour	0.95	0.00	0.00	−0.13
Cumulative variance	0.50	0.66	0.77	0.85

### Subanalysis of longitudinal change in depression and anxiety

**Figure 3:**
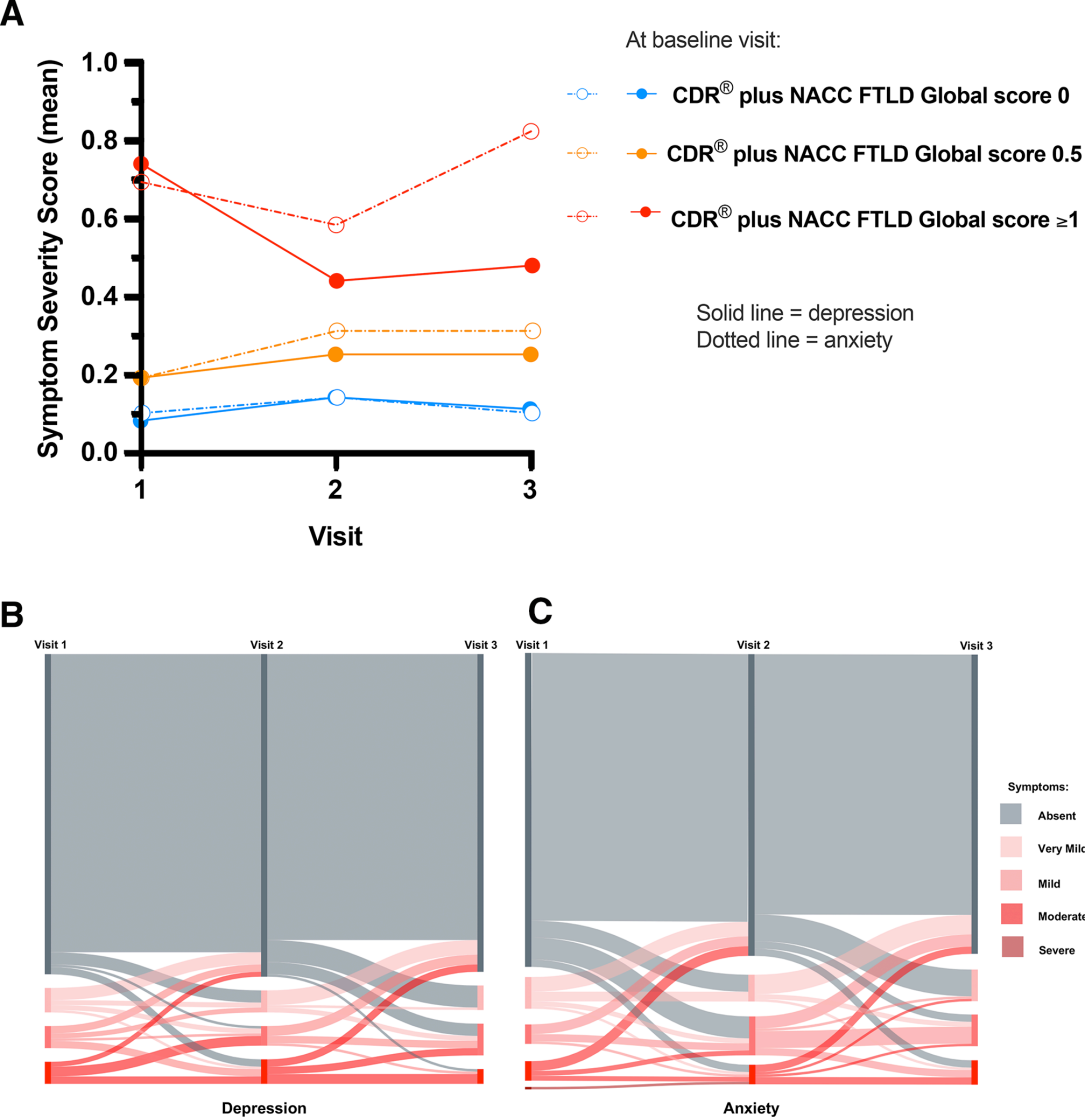
Longitudinal change in depression and anxiety in asymptomatic (CDR plus NACC FTLD global score of 0), prodromal (0.5) and symptomatic (≥1) mutation carriers: (A) mean severity of depression (solid line) and anxiety (dotted line) within all carriers; (B, C) Sankey diagrams showing individual change in depression (B) and anxiety (C) scores. CDR, Clinical Dementia Rating; NACC FTLD, National Alzheimer’s Coordinating Center Behaviour and Language Domains.

In the PCA, depression and anxiety consistently loaded together, often with symptoms likely to be secondary to these affective disorders such as impaired sleep and irritability/lability. In order to further consider whether affective symptoms should be included or excluded from any rating scale the longitudinal change was assessed. Within the combined mutation carrier group, and stratifying by CDR plus NACC FTLD, 55.6% had depression (mean severity 0.7 (0.8)) and 51.9% had anxiety (0.7 (0.9)) at visit one in the symptomatic (≥1) group, while at visit two 37.0% had depression (mean severity 0.4 (0.7)) and 44.4% had anxiety (0.6 (0.8)) and at visit three 40.7% had depression (mean severity 0.5 (0.7)) and 40.7% had anxiety (0.8 (0.8)). For the prodromal (0.5) group, 25.0% had depression (mean severity 0.2 (0.4)) and 25.0% had anxiety (0.2 (0.4)) at visit one, while at visit two 12.5% had depression (mean severity 0.3 (0.7)) and 31.3% had anxiety (0.3 (0.6)), and at visit three 25.0% had depression (mean severity 0.3 (0.5)) and 25.0% had anxiety (0.3 (0.6)). Finally, for the asymptomatic (0) group 7.8% had depression (mean severity 0.1 (0.3)) and 10.3% had anxiety (0.1 (0.4)) at visit one, while at visit two 12.9% had depression (mean severity 0.1 (0.4)) and 15.5% had anxiety (0.1 (0.4)), and at visit three 12.1% had depression (mean severity 0.1 (0.3)) and 12.1% had anxiety (0.1 (0.3)). Figure 3A highlights the change in mean severity of anxiety and depression within each group over time (with online supplemental figure 1 showing the same data for each of the genetic groups), while figure 3B, C shows the individual fluctuation in depression (3b) and anxiety (3c) symptoms over time.

### Rating scale analysis

**Figure 4:**
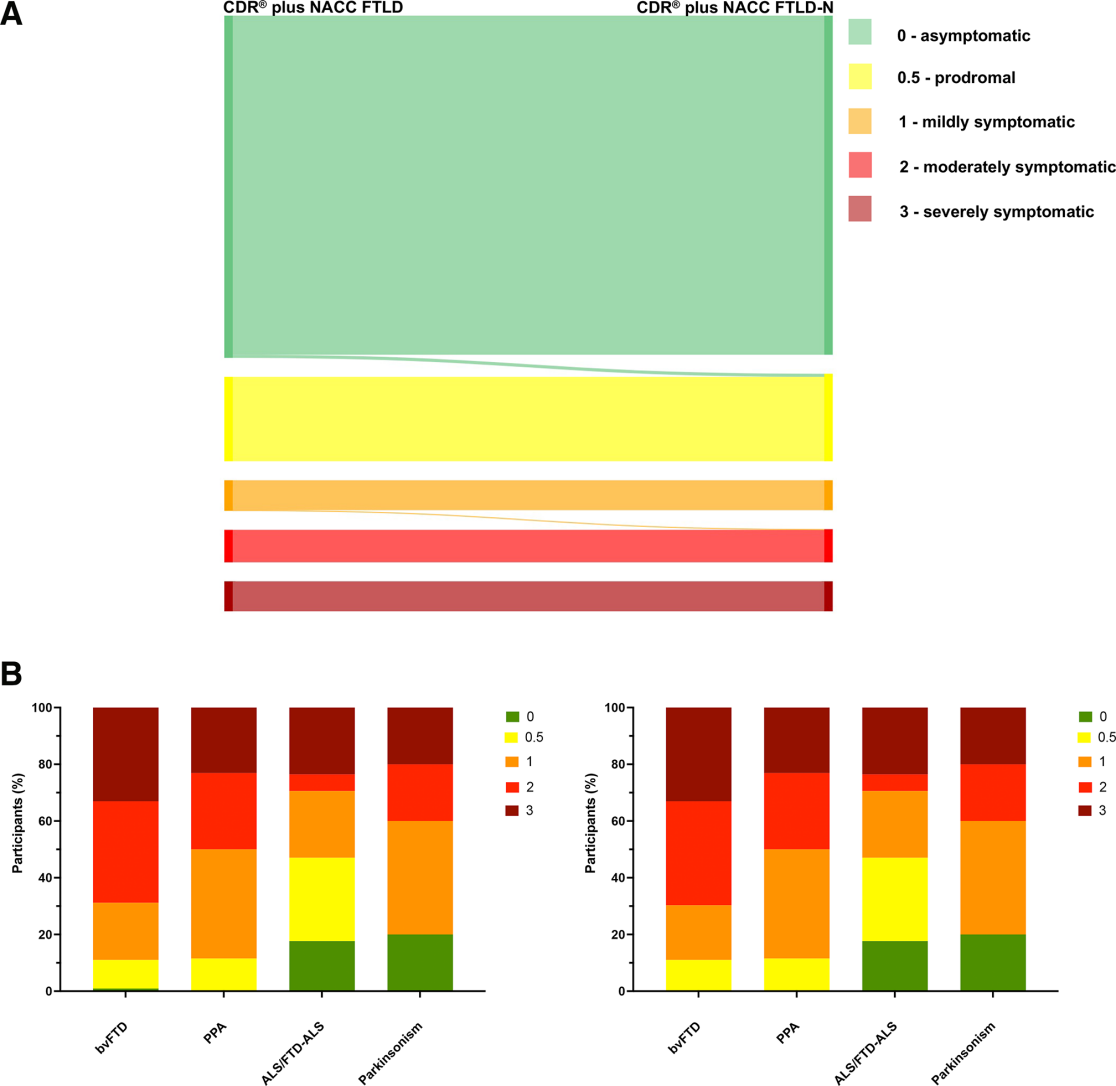
Comparison of the standard CDR plus NACC FTLD with a new CDR plus NACC FTLD plus Neuropsychiatric Score (CDR plus NACC FTLD-N). The top figure shows the change in global score in individual participants (five participants moved from 0 to 0.5 and 1 participant from 1 to 2). The bottom figure shows the percentage of symptomatic participants with a particular CDR score (left shows standard CDR plus NACC FTLD, right shows the change with the new CDR plus NACC FTLD-N). bvFTD, behavioural variant frontotemporal dementia; CDR, Clinical Dementia Rating; NACC FTLD, National Alzheimer’s Coordinating Center Behaviour and Language Domains; PPA, Primary Progressive Aphasia.

In light of the above results, we first investigated adding a neuropsychiatric module to the CDR plus NACC FTLD rating scale consisting only of the psychosis symptoms (hallucinations and delusions), excluding both affective symptoms and the other symptoms which loaded with the core behavioural features. We termed this the CDR plus NACC FTLD-N. This scale was positively correlated with the original CDR plus NACC FTLD (r=0.992, p<0.001). The new scale led to a number of participants (0.6%) now being considered prodromal who had previously been asymptomatic on the CDR plus NACC FTLD, as well as symptomatic participants with a bvFTD diagnosis now being considered more severely affected (figure 4).

**Figure 5:**
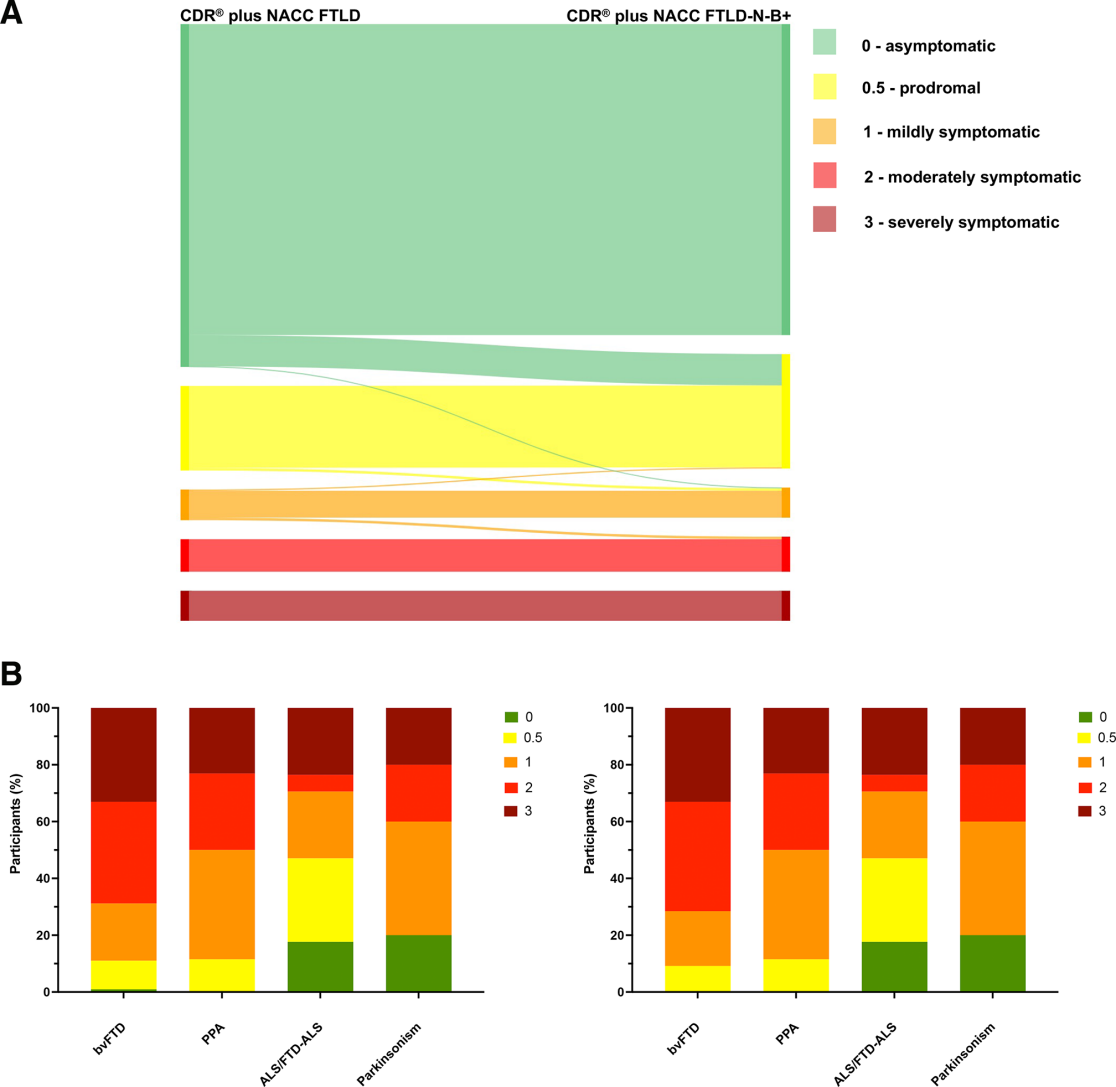
Comparison of the standard CDR plus NACC FTLD with the CDR plus NACC FTLD-N-B+. The top figure shows the change in global score in individual participants (50 participants moved from 0 to 0.5, 1 participant from 0 to 1, 4 participants from 0.5 to 1, 2 participants from 1 to 0.5 and 4 participants from 1 to 2). The bottom figure shows the percentage of symptomatic participants with a particular CDR score (left shows standard CDR plus NACC FTLD, right shows the change with the new CDR plus NACC FTLD-N-B+). CDR, Clinical Dementia Rating; FTD, behavioural variant frontotemporal dementia; NACC FTLD, National Alzheimer’s Coordinating Center Behaviour and Language Domains.

Second, we investigated a change to the behavioural module of the CDR plus NACC FTLD by (1) incorporating the non-psychosis and non-affective symptoms included in the GENFI neuropsychiatric symptoms scale and (2) generating an Algorithm-based Behaviour score from each of the individual behavioural symptoms (rather than using a Global Behaviour Score; see online supplemental table 4 for how this was generated). We termed this the CDR plus NACC FTLD-N-B+. In general, the Algorithm-based Behaviour Score led to fewer people being scored as 0 (68.4%, compared with 77.9% using the Global Behaviour Score), and more people being scored as 0.5 (very mild symptoms; 14.3%, compared with 6.4% using the Global Behaviour Score) (online supplemental figure 2). Including this within the CDR plus NACC FTLD alongside the new neuropsychiatric module also leads to more participants being considered prodromal (6% of people were asymptomatic on the CDR plus NACC FTLD but prodromal on the CDR plus NACC FTLD-N-B+) (figure 5) as well as clinically judged symptomatic participants being considered as more severe on the new scale, particularly those with a diagnosis of bvFTD (figure 4). Analysis of individual genetic mutation groups shows most of this change is in *C9orf72* mutation carriers (online supplemental figure 3).

## Discussion

This study has shown that neuropsychiatric features are common in genetic FTD and can occur early on in the disease process many years before people receive a clinical diagnosis. As such, they should be incorporated into any comprehensive clinical rating scale of FTD. However, the results here show that some of the neuropsychiatric symptoms can be variable, and careful thought is required in terms of which features are included in any scale. A PCA suggested that there are three main groups of neuropsychiatric symptoms: affective symptoms of anxiety and depression, ‘psychotic’ symptoms of hallucinations and delusions, and a set of ‘other’ symptoms including agitation, irritability and hypersexuality that loaded with the core behavioural features of bvFTD. While anxiety and depression are prominent symptoms in mutation carriers, they are also common in controls and fluctuate longitudinally over time suggesting they should be excluded from any scale. Furthermore, the association of the ‘other’ symptoms with the behavioural features of FTD suggest they should be considered as part of the ‘behavioural’ component of the scale rather than as a separate module. However, the third component including visual and auditory hallucinations and delusions separated out from the other features and was distinct to any current module in clinical FTD scales, particularly within the CDR plus NACC FTLD, suggesting this component should form a new neuropsychiatric module to specifically capture this element of the FTD phenotype. Adding this module to the CDR plus NACC FTLD (called here the CDR plus NACC FTLD-N) resulted in people previously being considered as asymptomatic now being considered prodromal, and in general people being rated as more severe, highlighting the importance of including psychotic symptoms in clinical rating scales of FTD.

### Frequency and severity of symptoms

Neuropsychiatric features were present in almost half the cohort studied, more commonly in those who were judged as symptomatic, but also occurring to a lesser extent prodromally (CDR plus NACC FTLD global score 0.5) and in some people at an asymptomatic stage (CDR plus NACC FTLD global score 0). Anxiety, depression and impaired sleep were the most common symptoms in mutation carriers. The presence of affective symptoms has been previously reported in symptomatic FTD, for example, a systematic review suggested a prevalence of 7%–69% of depression in FTD in general, and of 19%–63% of anxiety in bvFTD; the frequency in our study of 40.3% (depression) and 49.7% (anxiety) fits well within this range. However, such symptoms were also common in our controls with 12.9%, 15.5% and 13.2% reporting symptoms of depression, anxiety and impaired sleep, respectively. The prevalence of these conditions is around 5% for depression, 7% for anxiety and up to 30% for sleep problems in European countries, suggesting potentially higher rates in our cohort of controls compared with the general population (with the caveat that in GENFI we are measuring symptoms rather than making specific mental health diagnoses).^23 24^ The increased prevalence of affective disorders in mutation-negative family members has been poorly studied in genetic FTD but is a well-known phenomenon in neurogenetic disorders and can relate to multiple issues including survivor guilt in those who test negative within the family.

Of the mutation groups studied, *C9orf72* expansion carriers had the highest frequency of neuropsychiatric symptoms, followed by *MAPT* then *GRN* mutation carriers. Many studies have shown a close link between *C9orf72* expansion carriers and the presence of neuropsychiatric symptoms, both early on as a presenting feature and during the course of the disease.^25–28^ Moreover, *C9orf72* mutations are the most common genetic cause of ALS,^29 30^ with previous studies showing an association of ALS with early neuropsychiatric disturbances independent of other behavioural symptoms.^31^

Although less common than affective symptoms, ‘psychosis’ features, that is, hallucinations and delusions occur in up to a quarter of symptomatic patients, and more frequently in *C9orf72* than *GRN* and *MAPT* mutation carriers. This is consistent with prior studies showing they occur commonly in *C9orf72* mutation carriers,^6^ although not uniquely and can be present in the other genetic groups.^7 32^ In the current study, hallucinations and delusions were present only infrequently in *C9orf72* mutation carriers in the presymptomatic period, increasing in frequency when entering the symptomatic phase.^33^ Psychosis-type symptoms were least frequent and mildest in *MAPT* mutation carriers, although were still present, suggesting that these symptoms are not pathognomonic of a particular genetic subtype.

The only symptom that was more frequent in *MAPT* mutation carriers compared with the other groups was altered sense of humour. This has been poorly studied but has been associated with temporal lobe atrophy and *MAPT*-related FTD in one prior study.^34^

### Principal component analysis

PCA identified three main groups of symptoms as hypothesised. Importantly, this suggests that one cannot use a single neuropsychiatric score within a rating scale that incorporates all of the psychotic, affective and other neuropsychiatric symptoms. Combined with the depression/anxiety subanalysis, the results from this study suggest that a single neuropsychiatric score consisting of features of psychosis (ie, hallucinations and delusions) will be necessary in any future clinical rating scale.

Depression and anxiety not only formed a separate group to other neuropsychiatric symptoms, but also fluctuated longitudinally. These two factors combined with the relatively common occurrence in non-carriers support the argument for excluding depression and anxiety from global CDR scoring. There are multiple potential reasons for this fluctuation: the presence of depression and/or anxiety may coincide with life events including recovery with therapy or medication, a change in insight as the disease progresses, biological processes associated with the disease process, heightened anxiety/depression as individuals approach the age at onset of their relatives and subsequent fall in these symptoms if they pass this age, and a disease-related increase in anxiety as mutation carriers get older and more symptomatic from other affected domains.

Behavioural symptoms within the consensus diagnostic criteria for bvFTD^1^ are currently included in the CDR plus NACC FTLD and have often been viewed as separate from neuropsychiatric features. Nonetheless, multiple studies have highlighted the presence of both behavioural and neuropsychiatric symptoms in FTD and interestingly up to half of individuals with bvFTD may initially be given a psychiatric diagnosis.^2^ The loading of some of the neuropsychiatric symptoms (aberrant motor behaviour, euphoria/elation, altered sense of humour, irritability/lability, agitation/aggression and hypersexuality) with the core behavioural features of bvFTD suggests these should be considered when thinking about the behavioural component of any clinical rating scale rather than being included in a separate neuropsychiatric module.

Interestingly, the symptom of hyperreligiosity was distinct from the other three groups. This symptom has been described in association with temporal lobe deficits, particularly of the right hemisphere, and can be seen in right temporal lobe epilepsy^35^ as well as in people with right temporal lobe variant FTD.^36^ It is unclear why this does not associate with the other symptoms in the PCA, but in people with right temporal lobe variant FTD, the majority of the other symptoms are cognitive, for example, topographical memory loss and prosopagnosia, rather than behavioural, and it may be that the unique neuroanatomical association separates it from the other neuropsychiatric features. A further important point to note is that in the main analyses, tactile hallucinations (which are rare in FTD) had a negative loading with hyperreligiosity, the reason for which is unclear.

Overall, the PCAs and depression/anxiety subanalyses within the individual genetic mutation groups mirrored the findings when all mutation carriers were studied together, supporting the use of the same approach for all FTD individuals regardless of the genetic mutation, that is, a neuropsychiatric module consisting of ‘psychotic’ features, consideration of the ‘other’ neuropsychiatric features when scoring the behavioural domain, and exclusion of affective symptoms from the scale.

### A new neuropsychiatric module to add to the CDR plus NACC FTLD

Inclusion of the neuropsychiatric score into the CDR plus NACC FTLD (CDR plus NACC FTLD-N) led to more people being considered at higher disease stages than the original CDR, including some people being considered prodromal who were previously considered asymptomatic. In people clinically judged to be symptomatic the change in stage was mainly seen in those with bvFTD and minimal change in those with other clinical phenotypes, consistent with prior studies showing that while neuropsychiatric symptoms are seen in PPA and parkinsonian syndromes they are less common.

We also investigated a second addition to the CDR plus NACC FTLD, incorporating a version of the behavioural score that required consideration of multiple different symptoms including not only the core bvFTD symptoms but also the ‘other’ symptoms from the neuropsychiatric feature list. This also led to a general increase in disease stage severity, suggesting that perhaps clinicians are not considering the whole spectrum of behavioural features in FTD when scoring the behavioural module in the CDR plus NACC FTLD.

It is likely that these two additions to the CDR plus NACC FTLD lead to improved accuracy of rating disease stage in FTD and bring us closer to optimising the rating scale for use in therapeutic trials where precise scoring of an individual’s disease severity is important so that treatment responses can be more representative of their real-life experience with FTD.

### Limitations

Although this study represents one of the largest familial FTD cohorts, numbers become smaller in each group as they are stratified, particularly in longitudinal analyses, such as performed for depression and anxiety.

We had included symptoms in the GENFI neuropsychiatric symptom scale that had been previously reported in patients with FTD. However, reports of other symptoms such as anhedonia^37^ that have been more recently recognised as part of the FTD spectrum were not included.

When considering depression and anxiety we did not record the effect of therapeutic interventions on symptom severity such as antidepressants or psychological therapies. This did not allow us to measure their effect on the longer-term fluctuation in symptoms, and future studies should address this limitation.

All of the neuropsychiatric symptoms were recorded as being present or absent through the use of the GENFI symptom scale. While this is helpful for our current focus on improving clinical rating scales, it is important to note that this is distinct from making formal clinical diagnoses according to standard criteria of depression, anxiety, psychosis, etc, and therefore, may not be a true representation of the prevalence. Nonetheless, there are also potential reasons why neuropsychiatric symptoms may be underrepresented in GENFI as participants with active affective or psychotic disorders may not be able to (or want to) take part in an ongoing observational research study.

Lastly, although each rater received training in use of the neuropsychiatric symptom scales, now that its use is established it will be important in future studies to formally assess both intra- and inter-rater reliability.

In summary, neuropsychiatric symptoms commonly occur in individuals with FTD in all the main genetic mutation groups, including in those classed as ‘asymptomatic’. The failure of the current CDR plus NACC FTLD scale to account for psychotic symptoms runs the risk of adopting inaccurate tools to identify the disease staging and treatment response in individuals enrolled in therapeutic studies. This study suggests an initial step to rectifying this issue.

## Data Availability

Data are available on reasonable request. Some data are available on reasonable request after review by the GENFI Data Access Committee. Email is genfi@ucl.ac.uk.
