# Supplementary material for: Neuropsychiatric symptoms in genetic frontotemporal dementia: developing a new module for Clinical Rating Scales
Source: J Neurol Neurosurg Psychiatry. 2023 Jan 10;94(5):357–68. doi: 10.1136/jnnp-2022-330152 (PMC10176351; doi:10.1136/jnnp-2022-330152)

Supplementary Table 1. Neuropsychiatric symptoms included in the GENFI Symptom Scales in the History assessment. Scoring of each symptom is on a scale similar to the CDR i.e. 0 (absent), 0.5 (very mild/questionable), 1 (mild), 2 (moderate), and 3 (severe).

		Questionable/ Very mild	Mild	Moderate	Severe
0	**Neuropsychiatric**	Questionable neuropsychiatric symptoms.	Mild but definite neuropsychiatric symptoms.	Moderate neuropsychiatric symptoms.	Severe neuropsychiatric symptoms.
1	**Visual hallucinations**<br>Sees things that are not there	Some hallucinations may have occurred but are of questionable significance	Hallucinations are present but harmless and cause little distress for the patient.	Hallucinations are distressing and are disruptive to the patient.	Hallucinations are very disruptive and a major source of behavioural disturbance. PRN medications may be required to control them.
2	**Auditory hallucinations**<br>Hears voices	Some hallucinations may have occurred but are of questionable significance.	Hallucinations are present but harmless and cause little distress for the patient.	Hallucinations are distressing and are disruptive to the patient.	Hallucinations are very disruptive and a major source of behavioural disturbance. PRN medications may be required to control them.
3	**Tactile hallucinations**<br>Feels things that are not there	Some hallucinations may have occurred but are of questionable significance.	Hallucinations are present but harmless and cause little distress for the patient.	Hallucinations are distressing and are disruptive to the patient.	Hallucinations are very disruptive and a major source of behavioural disturbance. PRN medications may be required to control them.
4	**Delusions**<br>Paranoia, fear of harm, fear of theft, fear of abandonment, delusional misidentification	Some delusions may have occurred but are of questionable significance.	Delusions present but seem harmless and produce little distress in the patient.	Delusions are distressing and disruptive.	Delusions are very disruptive and are a major source of behavioural disruption. If PRN medications are prescribed, their use signals marked severity.
5	**Depression**<br>Low mood, tearful, talks about deserving punishment, talks about dying	Some features of depression may be present but are not distressing.	Depression is distressing but usually responds to redirection or reassurance.	Depression is distressing; depressive symptoms are spontaneously voiced by the patient and difficult to alleviate.	Depression is very distressing and a major source of suffering for the patient.
6	**Anxiety**<br>Feels shaky or tense, worried about planned events, phobic avoidance, separation anxiety	Some features of anxiety may be present but are not distressing.	Anxiety is distressing but usually responds to redirection or reassurance.	Anxiety is distressing, anxiety symptoms are spontaneously voiced by the patient and difficult to alleviate.	Anxiety is very distressing and a major source of suffering for the patient.

7	**Irritability/Lability**<br>Bad temper, rapid changes in mood, sudden flashes of anger, impatient, argumentative	Some irritable or labile behaviour may have occurred but of questionable significance.	Irritability or lability is notable but usually responds to redirection or reassurance.	Irritability and lability are very evident and difficult to overcome by the caregiver.	Irritability and lability are very evident; they usually fail to respond to any intervention by the caregiver, and they are a major source of distress.
8	**Agitation/Aggression**<br>Stubborn, unco-operative, hard to handle, slams doors, throws things, hits or harms others	Some agitated or aggressive behaviour may have occurred but of questionable significance.	Agitation is disruptive but can be managed by redirection or reassurance.	Agitation is disruptive and difficult to redirect or control.	Agitation is very disruptive and a major source of difficulty; there may be a threat of personal harm.
9	**Euphoria/Elation**<br>Feels too good or too happy, grandiose, childish behaviour	Some euphoria or elated behaviour may have occurred but of questionable significance.	Elation is notable to friends and family but is not disruptive.	Elation is notably abnormal, and disruptive.	Elation is very pronounced.
10	**Aberrant motor behaviour**<br>Paces without purpose, repeatedly dresses or undresses, excessively fidgety	Some aberrant motor behaviour may have occurred but of questionable significance.	Abnormal motor activity is notable but produces little interference with daily routines.	Abnormal motor activity is very evident; can be overcome by the caregiver.	Abnormal motor activity is evident, usually fails to respond to any intervention by the caregiver and is a major source of distress.
11	**Hypersexuality**<br>Unusual or excessive sexual behaviour	Some unusual or excessive sexual behaviour may have occurred but of questionable significance.	Sexual behaviour is disruptive but can be managed by redirection or reassurance.	Sexual behaviour is disruptive and difficult to redirect or control.	Sexual behaviour is very disruptive and a major source of difficulty.
12	**Hyperreligiosity**<br>Increased or unusual religious experiences or feelings	Possible increased or unusual religious experiences may have occurred but are of questionable significance.	Increased or unusual religious experiences are present but seem harmless and produce little distress in the patient.	Increased or unusual religious experiences may be distressing and disruptive.	Increased or unusual religious experiences are very disruptive and are a major source of behavioural disruption.
13	**Impaired sleep**<br>Difficulty sleeping at night, excessive sleep during the day	Possible impaired sleep but of questionable significance.	Sleep is notably impaired but produces little interference with daily routines (minimal daytime sleepiness).	Sleep very impaired during the night and may sleep regularly the day.	Extremely poor sleep; may be distressed during the night; partner's sleep markedly disturbed.
14	**Altered sense of humour**<br>Change in sense of humour, finds things funny which others don't	Sense of humour possibly altered but of questionable significance.	Mild but definite change in sense of humour. May finds things funny which others don't.	Sense of humour completely different from previously (e.g. may prefer more physical or 'slapstick' humour)	Complete loss of sense of humour.

Supplementary Table 2. Severity of neuropsychiatric symptoms in controls and mutation carriers. Scores are shown as mean (standard deviation). Bold items are significantly different to controls using linear regression ($p < 0.05$). Other differences are shown as [a]significantly impaired compared to GRN, [b]significantly impaired compared to MAPT and [c]significantly impaired compared to C9orf72 using logistic regression ($p < 0.05$).

	Controls	All mutation carriers			*C9orf72*			*GRN*			*MAPT*		
		CDR 0	CDR 0.5	CDR 1+	CDR 0	CDR 0.5	CDR 1+	CDR 0	CDR 0.5	CDR 1+	CDR 0	CDR 0.5	CDR 1+
Visual hallucinations	0.00 (0.03)	0.01 (0.12)	**0.04 (0.15)**	**0.17 (0.47)**	0.02 (0.19)	0.04 (0.18)	**0.26 (0.58)**[a]	0.00 (0.00)	0.03 (0.12)	0.10 (0.36)	0.00 (0.00)	0.04 (0.13)	0.06 (0.22)
Auditory hallucinations	0.01 (0.12)	0.00 (0.06)	0.00 (0.00)	**0.17 (0.53)**	0.01 (0.09)	0.00 (0.00)	**0.31 (0.71)**	0.00 (0.00)	0.00 (0.00)	0.06 (0.21)	0.00 (0.00)	0.00 (0.00)	0.00 (0.00)
Tactile hallucinations	0.00 (0.00)	0.00 (0.03)	0.01 (0.08)	**0.07 (0.38)**	0.00 (0.05)	0.03 (0.11)	**0.14 (0.53)**	0.00 (0.00)	0.00 (0.00)	0.00 (0.00)	0.00 (0.00)	0.00 (0.00)	0.02 (0.10)
Delusions	0.01 (0.07)	0.01 (0.07)	0.01 (0.06)	**0.32 (0.65)**	0.00 (0.05)	0.00 (0.00)	**0.51 (0.81)**[ab]	0.00 (0.00)	0.00 (0.00)	**0.13 (0.36)**	0.02 (0.14)	0.04 (0.13)	0.16 (0.47)
Depression	0.12 (0.39)	0.09 (0.33)	**0.30 (0.52)**	**0.48 (0.73)**	0.09 (0.33)	0.26 (0.58)	**0.53 (0.81)**	0.07 (0.27)	**0.29 (0.38)**	**0.45 (0.64)**	0.14 (0.44)	0.43 (0.62)	**0.40 (0.68)**
Anxiety	0.15 (0.42)	0.10 (0.35)	**0.33 (0.47)**	**0.57 (0.73)**	0.08 (0.32)	0.30 (0.48)	**0.62 (0.78)**	0.12 (0.41)	**0.34 (0.49)**	**0.49 (0.67)**	0.08 (0.26)	0.39 (0.45)	**0.58 (0.75)**
Irritability/lability	0.09 (0.30)	0.03 (0.13)	**0.21 (0.42)**	**0.60 (0.79)**	0.03 (0.11)	0.23 (0.42)	**0.71 (0.84)**	0.02 (0.12)	0.19 (0.44)	**0.42 (0.68)**	0.04 (0.17)	0.18 (0.37)	**0.68 (0.83)**
Agitation/aggression	0.02 (0.17)	0.01 (0.06)	0.07 (0.20)	**0.31 (0.55)**	0.01 (0.07)	0.08 (0.25)	**0.37 (0.61)**	0.00 (0.04)	0.08 (0.19)	**0.18 (0.34)**	0.01 (0.07)	0.00 (0.00)	**0.42 (0.67)**
Euphoria/elation	0.00 (0.03)	0.01 (0.05)	**0.04 (0.18)**	**0.34 (0.66)**	0.01 (0.08)	0.04 (0.18)	**0.42 (0.76)**	0.00 (0.00)	0.03 (0.18)	**0.17 (0.39)**	0.00 (0.00)	0.07 (0.18)	**0.44 (0.75)**
Aberrant motor behaviour	0.01 (0.07)	0.00 (0.06)	0.03 (0.12)	**0.51 (0.84)**	0.00 (0.00)	0.03 (0.11)	**0.60 (0.92)**	0.00 (0.00)	0.05 (0.15)	**0.35 (0.68)**	0.02 (0.14)	0.00 (0.00)	**0.56 (0.89)**
Hypersexuality	0.00 (0.03)	0.00 (0.00)	0.02 (0.09)	**0.21 (0.57)**	0.00 (0.00)	0.04 (0.14)	**0.24 (0.59)**	0.00 (0.00)	0.00 (0.00)	**0.13 (0.43)**	0.00 (0.00)	0.00 (0.00)	0.26 (0.72)
Hyperreligiosity	0.00 (0.00)	0.00 (0.03)	0.01 (0.08)	**0.14 (0.52)**	0.00 (0.00)	0.00 (0.00)	**0.18 (0.60)**	0.00 (0.04)	0.03 (0.12)	0.05 (0.20)	0.00 (0.00)	0.00 (0.00)	0.24 (0.71)
Impaired sleep	0.13 (0.40)	0.06 (0.27)	0.25 (0.49)	**0.55 (0.76)**	0.02 (0.12)	0.24 (0.47)	**0.69 (0.87)**	0.07 (0.29)	0.31 (0.57)	**0.38 (0.57)**	0.12 (0.43)	0.14 (0.31)	**0.48 (0.70)**
Altered sense of humour	0.00 (0.04)	0.00 (0.03)	0.03 (0.14)	**0.60 (0.84)**	0.00 (0.00)	0.05 (0.20)	**0.52 (0.79)**	0.00 (0.04)	0.02 (0.09)	**0.47 (0.70)**	0.00 (0.00)	0.00 (0.00)	**1.12 (1.07)**[ac]

Supplementary Table 3. Principal component analysis of (a) neuropsychiatric symptoms and (b) combined neuropsychiatric and behavioural symptoms in *C9orf72*, *GRN* and *MAPT* mutation carriers. As there were no observations for tactile hallucination in the *GRN* group, and for auditory hallucinations in the *MAPT* group these variables were excluded from the PCA in these groups.

(a)

	C9orf72				*GRN*			*MAPT*			
**Component**	**1**	**2**	**3**	**4**	**1**	**2**	**3**	**1**	**2**	**3**	**4**
**Visual hallucinations**	0.00	0.89	0.27	0.00	0.49	0.71	0.00	0.76	-0.39	-0.49	-0.13
**Auditory hallucinations**	0.22	0.86	0.26	0.00	0.23	0.84	0.33	-	-	-	-
**Tactile hallucinations**	0.33	0.17	0.42	-0.81	-	-	-	-0.37	0.74	-0.43	-0.36
**Delusions**	0.41	0.74	0.33	0.00	0.76	0.25	0.18	0.00	0.00	0.85	0.27
**Depression**	0.00	0.20	0.83	-0.14	0.14	0.34	0.78	0.27	0.00	0.25	0.82
**Anxiety**	0.13	0.41	0.72	0.00	0.26	0.12	0.85	0.32	0.57	0.00	0.50
**Irritability/lability**	0.62	0.00	0.65	0.15	0.74	0.21	0.49	0.00	0.84	0.20	0.31
**Agitation/aggression**	0.66	0.00	0.61	0.14	0.85	0.16	0.35	0.56	0.68	-0.10	0.00
**Euphoria/elation**	0.84	0.23	0.17	0.00	0.69	0.58	0.00	0.21	0.21	0.87	0.00
**Aberrant motor behaviour**	0.68	0.55	0.00	0.00	0.69	0.40	0.21	0.88	0.14	0.18	0.25
**Hypersexuality**	0.76	0.11	0.18	-0.26	0.59	0.68	0.34	0.00	0.80	0.46	0.00
**Hyperreligiosity**	0.20	0.46	0.17	0.83	0.00	0.66	0.50	0.76	0.00	0.52	-0.18
**Impaired sleep**	0.32	0.39	0.69	0.00	0.34	0.16	0.72	-0.22	0.00	0.11	0.88
**Altered sense of humour**	0.76	0.49	0.00	0.27	0.39	0.76	0.20	0.58	0.18	0.61	0.33
**Cumulative variance**	**0.26**	**0.50**	**0.71**	**0.82**	**0.29**	**0.56**	**0.77**	**0.23**	**0.45**	**0.68**	**0.85**

(b)

	C9orf72				***GRN***				***MAPT***			
**Component**	**1**	**2**	**3**	**4**	**1**	**2**	**3**	**4**	**1**	**2**	**3**	**4**
**Visual hallucinations**	0.25	0.86	0.26	0.11	0.38	0.78	0.00	0.11	0.43	-0.26	-0.13	-0.84
**Auditory hallucinations**	0.41	0.81	0.22	0.10	0.25	0.74	0.30	0.50	-	-	-	-
**Tactile hallucinations**	0.32	0.15	0.42	-0.81	-	-	-	-	-0.42	0.85	-0.27	0.00
**Delusions**	0.47	0.66	0.35	0.00	0.68	0.29	0.32	-0.13	0.43	-0.11	0.22	0.71
**Depression**	0.00	0.22	0.83	-0.11	0.00	0.33	0.75	0.35	0.25	-0.13	0.83	0.00
**Anxiety**	0.16	0.40	0.72	0.00	0.15	0.00	0.86	0.19	0.22	0.45	0.62	0.00
**Irritability/lability**	0.61	0.00	0.65	0.00	0.45	0.41	0.67	-0.14	0.17	0.74	0.41	0.28
**Agitation/aggression**	0.67	-0.13	0.61	0.00	0.55	0.39	0.57	-0.27	0.54	0.69	0.00	-0.29
**Euphoria/elation**	0.82	0.00	0.20	-0.15	0.53	0.72	0.23	0.00	0.63	0.00	0.11	0.65
**Aberrant motor behaviour**	0.73	0.42	0.12	0.00	0.75	0.26	0.29	0.00	0.83	0.00	0.27	-0.28
**Hypersexuality**	0.70	0.00	0.23	-0.30	0.53	0.61	0.41	0.25	0.33	0.67	0.00	0.47
**Hyperreligiosity**	0.33	0.39	0.17	0.83	0.31	0.14	0.35	0.72	0.79	0.00	-0.11	0.00
**Impaired sleep**	0.32	0.33	0.70	0.00	0.41	0.00	0.69	0.20	0.00	0.00	0.82	0.25
**Altered sense of humour**	0.81	0.34	0.00	0.20	0.62	0.48	0.14	0.44	0.87	0.00	0.31	0.24
**Disinhibition**	0.79	0.37	0.27	-0.14	0.70	0.52	0.20	0.00	0.73	0.62	0.00	0.13
**Apathy**	0.74	0.37	0.38	0.11	0.80	0.25	0.19	0.35	0.86	0.00	0.25	0.00
**Loss of sympathy/empathy**	0.75	0.41	0.31	0.16	0.83	0.37	0.15	0.23	0.77	0.45	0.00	0.00
**Ritualistic/compulsive behaviour**	0.79	0.30	0.22	0.00	0.72	0.28	0.31	0.30	0.68	0.56	0.14	0.14
**Hyperorality and appetite changes**	0.83	0.38	0.11	0.13	0.77	0.20	0.35	0.26	0.88	0.00	0.24	0.20
**Poor response to social/emotional cues**	0.76	0.37	0.29	0.00	0.93	0.23	0.14	0.14	0.81	0.48	0.11	0.00
**Inappropriate trusting behaviour**	0.81	0.33	0.15	0.00	0.80	0.36	0.16	0.17	0.90	0.12	0.10	0.13
**Cumulative variance**	**0.39**	**0.56**	**0.73**	**0.81**	**0.37**	**0.55**	**0.73**	**0.82**	**0.40**	**0.59**	**0.71**	**0.82**

Supplementary Table 4. Methodology for calculating the Algorithm-based Behaviour Score.

Individual scores	Overall score
All 0	0
Maximum 0.5	0.5
Maximum > 0.5:	
Maximum 1, all others 0	0.5
Maximum 2 or 3, all others 0	1
Maximum occurs once, another rating > 0	One level < maximum
Maximum occurs > once	Maximum score

Supplementary Figure 1. Longitudinal change in severity of depression and anxiety in asymptomatic (CDR® plus NACC FTLD global score 0), prodromal (0.5) and symptomatic (≥1) *C9orf72, GRN* and *MAPT* mutation carriers. Solid lines represent depression severity scores and dotted lines represent anxiety severity scores.

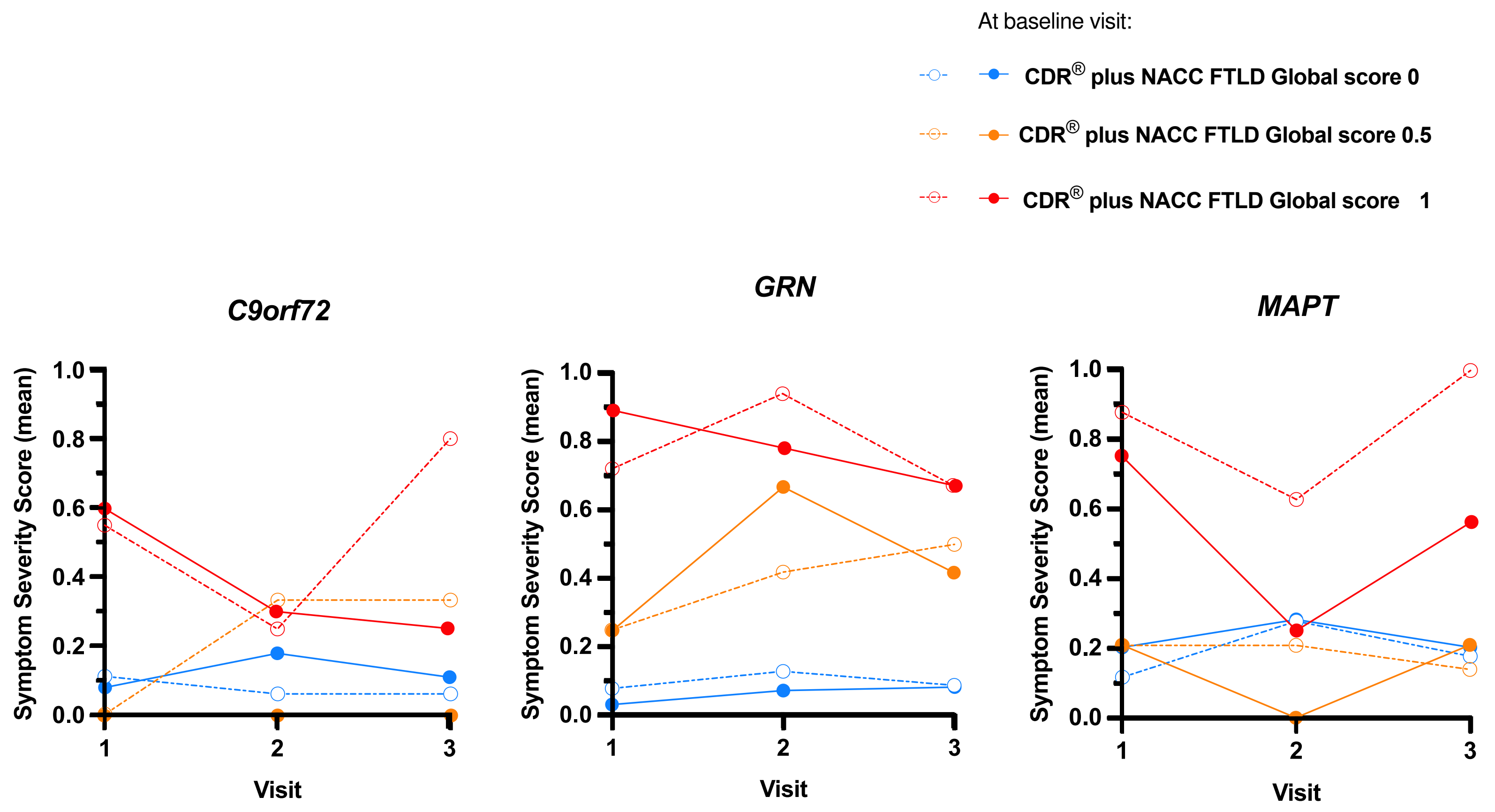

Supplementary Figure 2. Comparison of Global Behaviour Score and newly designed Algorithm-based Behaviour Score. Individual (left panel) and group (right panel) comparisons are shown.

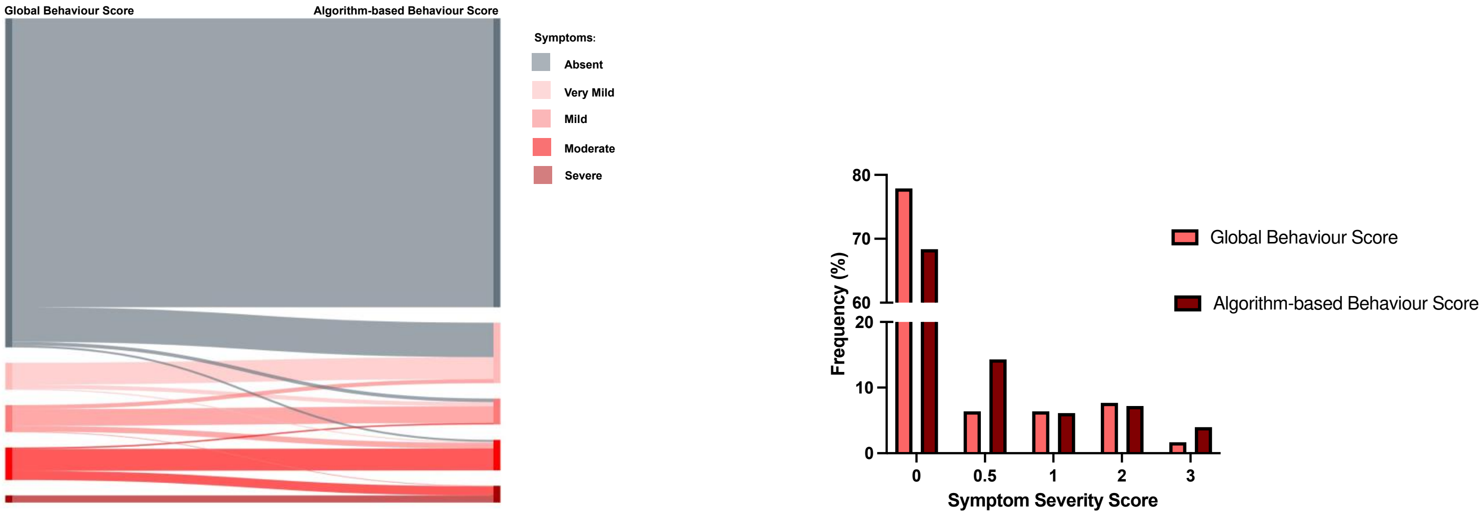

Supplementary Figure 3. Comparison of the standard CDR® plus NACC FTLD with a new CDR® plus NACC FTLD which adds in a new Neuropsychiatric Score and replaces the original NACC global Behaviour component with an algorithm-based Behaviour Score (CDR® plus NACC FTLD-N-B+) in genetic mutation carrier groups (*C9orf72*, *GRN* and *MAPT*) judged clinically to be symptomatic.

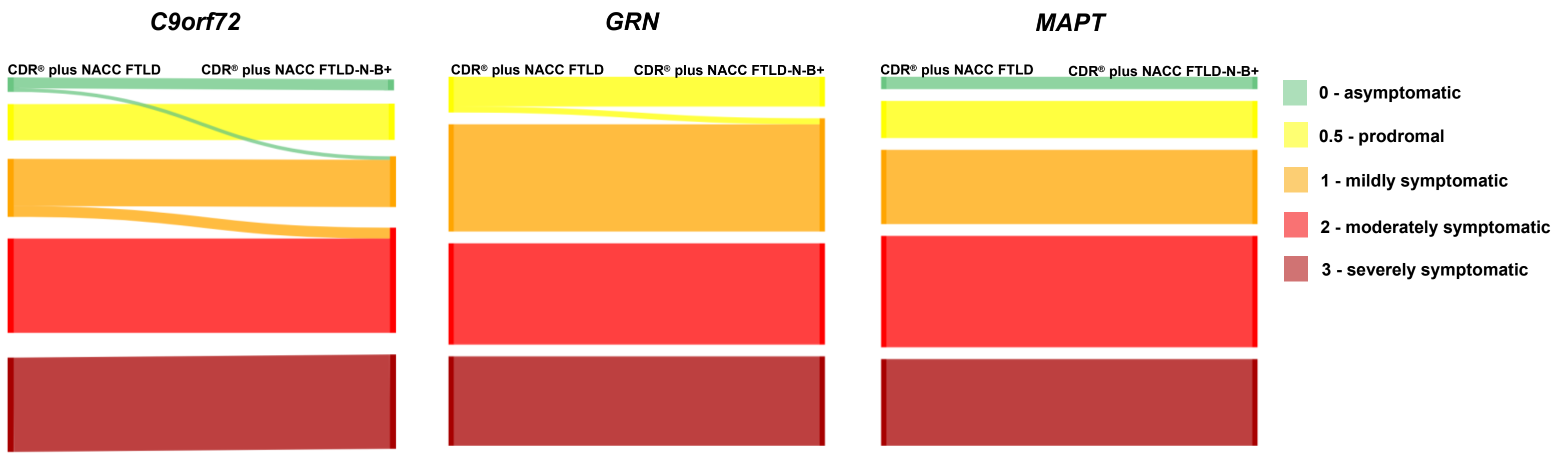

Supplement: Supplementary data [file jnnp-2022-330152supp001.pdf]
